# Challenges and limitations in using bacterial metabolites as immunomodulators

**DOI:** 10.3389/fcimb.2025.1535394

**Published:** 2025-01-29

**Authors:** Chinnashanmugam Saravanan, Nandana Karrath Gopinath, Raja Ganesan, Durairaj Thirumurugan

**Affiliations:** ^1^ Research & Development Division, MicroPros Lab Inc., Edmonton, AB, Canada; ^2^ School of Biotechnology, Amrita Vishwa Vidyapeetham, Kerala, India; ^3^ Department of Biotechnology, Faculty of Science and Humanities, SRM Institute of Science and Technology, Chennai, Tamil Nadu, India

**Keywords:** gut bacterial metabolites, microbiome, immunomodulators, metagenomics, synthetic biology

## Abstract

Harnessing the immunomodulatory potential of bacterial metabolites opens up exciting possibilities for treating various immune-related disorders. However, turning this potential into a reality presents significant challenges. This review investigates these challenges, focusing on discovery, production, characterization, stability, formulation, safety, and individual variability limitations. The limited bioavailability of many metabolites, as well as potential improvements along with the potential for off-target effects and the importance of precise targeting, are emphasized. Furthermore, the complex interactions between gut bacterial metabolites and the microbiome are investigated, highlighting the importance of personalized approaches. We conclude by discussing promising advances in metagenomics, metabolomics, synthetic biology, and targeted delivery systems, which hold out hope for overcoming these limitations and paving the way for the clinical translation of bacterial metabolites as effective immunomodulators.

## 1 Introduction

The wide range of small molecules that bacteria make due to their metabolic processes are called bacterial metabolites. These molecules are essential to many different biological interactions and activities. Although they are not directly involved in core metabolic pathways, secondary metabolites frequently support the competitiveness, adaptation, and survival of bacteria. Secondary metabolites are typically generated during particular stages of growth or in response to stress. It has been discovered that secondary metabolites contain antibacterial, anti-inflammatory, and anti-aging properties (Demain and Vaishnav, 2011). Antitumor, cholesterol-lowering, immunosuppressive, antiprotozoal, antihelminth, antiviral, and anti-ageing properties are some of these alternative uses. Bacterial-derived metabolites have the potential to be immunoactive agents, but there are several challenges associated with their use (Arun et al., 2022). The complex interactions between immune cells and microbial metabolites must be better understood to harness their immunomodulatory effects completely (Uchimura et al., 2018).

The potential for systemic side effects and the need to modulate the local microbiota further complicate using bacterial-derived metabolites as immune active agents (Garrett, 2020). The human gut harbors a vast population of microbes that produce secondary metabolites, which have been shown to influence human health by modulating metabolism, immune function, and the nervous system. The human gut microbiota and humans have a symbiotic relationship, which indicates that the metabolites these microorganisms produce, as well as their presence are essential to the host’s health (Afzaal et al., 2022). Gut dysbiosis directly and negatively affects host homeostasis because certain gut microorganisms are responsible for the bioavailability of certain metabolites (Krishnamurthy et al., 2023). Particularly, a few metabolites derived from the microbiota regulate populations of adaptive immune cells like Th17 and Treg cells. This influences immune regulation and plays a role in immune-related or immune-mediated diseases. Scaling up production for commercial use demands optimization of fermentation processes and improvements in yields, making it imperative to address these knowledge gaps and technical intricacies to successfully apply microbial metabolites across various industries (O’Brien and Wright, 2011). Moreover, the chemical complexity of microbial secondary metabolism presents challenges in studying and manipulating these metabolites effectively, requiring a deeper understanding of synthesis pathways and regulation. While bacterial derived metabolites hold promise as immunoreactive agents, further research is needed to overcome these challenges and fully exploit their therapeutic potential.

## 2 Immunomodulatory activities of bacterial metabolites

Common commensal microorganisms that can synthesize natural substances with anti-inflammatory and immunomodulatory effects include *Lactobacilli, Bifidobacteria, Bacteroides fragilis, E. coli*, and *Bacillus* sp. Pro-inflammatory cytokines like IL-1β, IL-6, and TNF-α are involved in pathological pain processes. Natural compounds from microorganisms have the potential to modulate cytokines and inhibit inflammation. These natural compounds including omega-3 fatty acids, cyclic peptides, and antimicrobial peptides offer anti-inflammatory effects that can be easily incorporated into diets without adverse effects (**Table 1**).

**Table 1:** Bacterial metabolic peptides and short-chain fatty acid as immunomodulators.

S. No	Metabolites/ Peptides	Bacteria	Immunomodulatory response	Reference
1	Nisin	*Lactococcus lactis*	Stimulation increased IL-1β, IL-6 production and the percentage of CD4^+^ CD8^+^ T cells in unstimulated leukocytes.	(Małaczewska et al., 2019)
2	Bifidococcin	*Bifidobacterium longum* sub sp. *infantis*	Enhanced levels of IL-8, IL-6, and the pro-inflammatory cytokine TNF-α amplify the inflammatory response.	(Javvadi et al., 2022)
3	Bifidocin LHA	*Bifidobacterium adolescentis*	Enhanced levels of IL-10 and IL-12 were induced, helping to mitigate damage caused by the inflammatory response.	(Mahdi et al., 2021)
4	Acidocin A	*Lactobacillus acidophilus*	The production of several inflammatory mediators (such as IL-6, TNFα, MIG/CXCL9, MCP-1/CCL2, MCP-3/CCL7, and MIP-1β) was induced, while the production of certain anti-inflammatory factors (including IL-1RA and MDC/CCL22) was inhibited.	(Antoshina et al., 2022)
5	Enterocin DD14	*Enterococcus faecalis*	The levels of pro-inflammatory IL-6 and IL-8 secreted in treated Caco-2 cells were significantly reduced with EntDD14	(Teiar et al., 2022)
Short-chain fatty Acid				
6	Butyric Acid	*Lacticaseibacillus paracasei* and *Lacticaseibacillus rhamnosus*	Butyrate inhibited pathogen-induced pro-inflammatory cytokines, particularly IL-8.	(Thananimit et al., 2022)
7	Butyric Acid	*Staphylococcus epidermidis*	Significant increase in the production of the pro-inflammatory cytokine IL-6.	(Keshari et al., 2019)
8	Butyric Acid	*Lactobacillus acidophilus* and *Bacillus subtilis*	FAM increased CD4+ T cells, SIgA+ cells, and SIgA production in the intestine, while also boosting the expression of IL-22, GPR43, GPR41, AhR, and HIF-1α.	(Xie et al., 2022)

Examples such as exopolysaccharides, peptide bacteriocin, polycyclic peptide bacteriocin (nisin), and short-chain fatty acids (SCFAs) are proposed for treating conditions like atherosclerosis, orthopedic postoperative infections, and mycobacterium tuberculosis infection (Jenab et al., 2020). Bacterial compounds are crucial in inducing immunomodulation and boosting the immune system. Lipoamino glycosides can improve immunotherapies by promoting antitumor immune cell activity (Colombani et al., 2019). Notable examples of immunomodulatory compounds include lipopolysaccharide and lipid A (Le Garrec, 1986; O’Brien and Wright, 2011). These compounds, particularly Lipid A and its analogs, exhibit immunomodulatory effects such as adjuvant activity, enhancing immune responses, and immunostimulation. Bacterial derived lipopolysaccharide and Lipid A are well-studied immunomodulatory substances; research is being done on synthetic versions. Additionally, synthetic derivatives of gram-negative bacterial cell walls activate immune responses, serving as immunoadjuvants to enhance vaccine effects. Furthermore, meta-MAMP (Microbe-Associated Molecular Patterns) like rapamycin and soraphen A can modulate T cell fate and function by targeting key metabolic pathways triggered during T cell activation. Recent research indicates that microbial products such as short-chain fatty acids and metabolites like retinoic acid may also play a significant role in controlling the functional specialization of Tregs (Hoeppli et al., 2015).

Cyclosporin A was initially found as a fungus-produced substance with antifungal properties. However, its discovery as an immunosuppressant led to its use in organ transplants. Although successful, newer and more potent immunosuppressants, FK-506 and rapamycin, were developed, offering better results with fewer side effects. These drugs interact with a specific protein inside cells, disrupting the signals that activate immune responses. Another immunosuppressant, mycophenolic acid, derived from an old antibiotic, has been developed for use after being initially discovered in 1896. It was found effective for treating psoriasis before being approved as an immunosuppressant (Demain, 1999). Microbial metabolites bidirectionally promote tolerance and immunity to effectively fight infection without developing inflammatory diseases. Numerous metabolites produced by bacteria that have immunomodulatory properties have been found. The actinobacteria *Streptomyces calvus*, are prolific sources of secondary metabolites with immunomodulatory effects on cytokine gene expression (Mahmoudi et al., 2016). Monophosphoryl lipid A, a safe lipidic derivative from bacteria, has been accepted as an adjuvant in humans and has demonstrated potential in enhancing immunotherapies (Colombani et al., 2019). Similar immunomodulatory agents are released by *Staphylococcus aureus* biofilm and planktonic cultures, encouraging leukocytes to generate cytokines and other immune responses (Sadowska et al., 2013). It has also been discovered that certain antibiotics, including spergualin, neothramycin, mazethramycin, aclacinomycin, and oxanosine, have immunomodulatory properties (Ishizuka et al., 1995). The polysaccharides, exopolysaccharides, capsular polysaccharides, selenium-exopolysaccharide, mannosyl‐oligosaccharides, glucooligosaccharides, oligosaccharides, and fructooligosaccharides offer immunomodulatory effects were summarized in **Table 2**.

**Table 2:** Summary of gut bacterial metabolites and their immunomodulatory response.

S. No.	Metabolites	Bacteria	Immunomodulatory response	Reference
1	Exopolysaccharides	*Bacillus licheniformis* and *Leuconostoc mesenteroides*	Enhanced secretion of the anti-inflammatory cytokine IL-10	(Kook et al., 2019)
2	Exopolysaccharides	*Lactobacillus casei*	Induce anti-inflammatory cytokine IL-10	(Górska et al., 2016)
3	Polysaccharides	*Lactobacillus rhamnosus*	Enhanced interleukin-10 (IL-10) and IL-12p70 levels	(Górska et al., 2014)
4	Exopolysaccharides	*Lacticasei bacillus rhamnosus*	Promote the secretion of NO, TNF-α, IL-1β, and IL-6.	(Chen et al., 2022)
5	Polysaccharides	*Bacillus velezensi*	Enhance the release of NO, TNF-α, IL-1β, and IL-6.	(Le et al., 2024)
6	Polysaccharides	*Lactobacillus paracasei*	Boosting the phagocytic function of macrophage cells	(Liu et al., 2022)
7	Exopolysaccharides	*Bifidobacterium longum*	Enhance the secretion of IFN-γ, IL-1β, and IL-6 in peripheral blood mononuclear cells	(Inturri et al., 2017)
8	Capsular Polysaccharides	*Lactobacillus fermentum*	Enhancing macrophage cell proliferation and phagocytic activity by increasing the secretion of IL-1β, IL-6, IL-10, and TNF-α	(Wang et al., 2023)
9	Selenium-exopolysaccharide	*Lactococcus lactis*	Enhanced phagocytic activity along with increased secretion of nitric oxide, IFN-γ, IL-1, IL-6, and IL-12	(Guo et al., 2013)
10	Exopolysaccharides	*Lactobacillus helveticus*	Promoting the production of NO and cytokines, and enhancing phagocytosis in macrophage cells	(You et al., 2020)
11	Oligosaccharides	*Leuconostoc lactis*	Decreased nitric oxide production, suppressed expression of iNOS, TNF-α, IL-1β, IL-6, IL-10, and inhibition of NF-κB signalling all indicate anti-inflammatory activity.	(Kim et al., 2021)
12	Mannosyl‐oligosaccharides	*Corynebacterium glutamicum*	The levels of cytokine TNF-α and interleukin-6 (IL-6) in macrophage cells were significantly increased.	(Liu et al., 2022)
13	Glucooligosaccharides	*Weissella cibaria*	Synergistically boosting nitric oxide production, synbiotics enhanced the expression of IL-1β, TNF-α, and IL-6 in macrophage cells.	(Jeong et al., 2023)
14	Fructooligosaccharides	*Bacillus subtilis*	FOSs decreased OVCAR-3 cell viability and proliferation, inducing IL-8 and TNF-α production and repressing ER-β genes.	(Magri et al., 2020)

## 3 Challenges and limitations of bacterial metabolites

### 3.1 Bioavailability

Harnessing bacterial metabolites as immunoreactive agents faces challenges in terms of bioavailability. The efficient transport of these compounds to target tissues is hampered by the local microbiota, systemic side effects, and physiological mucosal barriers (Xue et al., 2023). Furthermore, because bacterial metabolites are complex and varied, it is critical to identify their constituent parts to comprehend their immune-stimulatory capabilities (Puccetti et al., 2023). Additionally, since both can affect the host immune system and metabolism, it can be difficult to discriminate between microbial and host metabolites (Singh et al., 2014). Their production is hampered by the small amount of product that is recovered after fermentation and the laborious downstream processing required to extract the product (Castell et al., 2022). By identifying the fundamental metabolic processes involved in their synthesis, attempts are being undertaken to improve their production. For these metabolites to effectively promote health and have therapeutic applications, their bioavailability must be increased (Ganjoo et al., 2022). Derivatization, microencapsulation, nanoformulations, and bio-enhancers are a few strategies that have been investigated to boost these metabolites’ bioavailability (Sandhir et al., 2021). These methods have demonstrated potential for increasing the extracellular bacterial metabolites’ bioavailability and, thus, their therapeutic potential (Du et al., 2022). By addressing these issues, harnessing bacterial metabolites as immunoreactive agents can offer novel therapeutic strategies for immunotherapy, promoting therapeutic efficacy and reducing toxicity (Uchimura et al., 2018).

### 3.2 Potential toxicity

Microbial metabolites promote tolerance and immunity, but in pathogenic conditions, adverse effects have been observed. Bacterial toxins can influence the immune system, which can result in immunological responses to co-administered antigens or the inhibition of immune cell activation (Du et al., 2022). It has been discovered that the microbiota and their metabolites affect the toxicity and effectiveness of cancer immunotherapy (Kubickova et al., 2019). The gut microbiome has been linked to immune-related adverse effects of immune checkpoint inhibitors and may influence checkpoint inhibitors outcomes (Yanev and Stoyanova, 2018). Additionally, exposure to cyanobacterial metabolites, such as microcystins and cylindrospermopsin, can result in gastrointestinal symptoms and immune system effects (Donaldson and Williams, 2009).

Fluoranthene is an example of a bacterial secondary metabolite that is potentially harmful to the immune system (Modimola et al., 2022). Furthermore, it has been discovered that the metabolite 3-indole propionic acid has a negative immune-modulation impact. This finding may be useful for regulating the responses of cytotoxic T lymphocytes in autoimmune disorders, type I diabetes, and COVID-19 (Pittino et al., 2022). Moreover, it has been demonstrated that metabolites produced by the microbiota, such as trimethylamine N-oxide, tryptophan metabolites, and SCFAs, influence the immune system and may be useful targets for treatment in immunological-mediated dermatological disorders (Guijas et al., 2022).

### 3.3 Off-target effects

It has been demonstrated that bacterial metabolites influence the immune system through immunomodulation. For instance, it has been discovered that the metabolite 3-indole propionic acid contributes to T-cell exhaustion and immunosuppression (Guijas et al., 2022). Adenosine and itaconate, two other metabolites that have been identified as anti-inflammatory, can aid in limiting the local damage brought on by inflammation (Urso and Prince, 2022). The pathophysiology of inflammatory illnesses, such as multiple sclerosis, has been linked to the gut microbiota and its metabolites. This is because they have an impact on immune cell priming and facilitate the development of regulatory T-cells (Du et al., 2022). However, it is important to note that bacterial metabolites can have off-target limitations on the immune system. For instance, it has been demonstrated that the bacterial metabolite n-butyrate inhibits dendritic cell maturation and decreases its capacity to activate T cells (Saemann et al., 2002). Developing techniques for treatment and focusing on the microbiome to improve immune responses in a variety of disorders requires an understanding of the limitations and effects of bacterial metabolites on the immune system (Haase et al., 2018).

### 3.4 Other challenges

**Figure 1:**
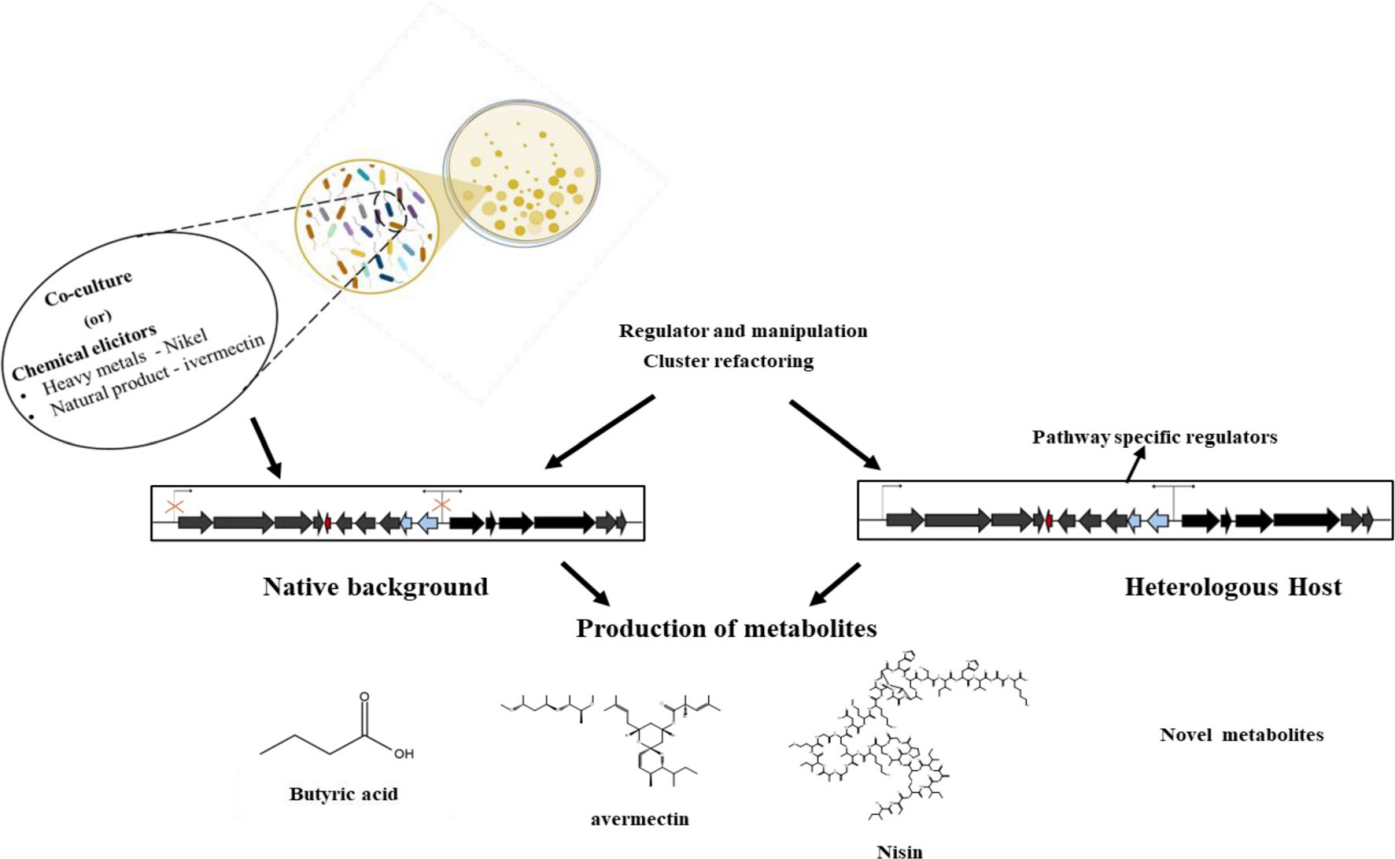
Cryptic biosynthetic gene clusters. Source (Zhang et al., 2019).

The production of bioactive compounds primarily results from the activation of cryptic gene clusters, which are normally dormant. Therefore, the expression of these clusters could prove beneficial in harnessing the chemical diversity found in microorganisms. The need to develop techniques to comprehend the intricate mechanisms of cryptic genes and their relationship to the production of bioactive compounds, the lack of knowledge regarding enhancing the production of bioactive secondary metabolites, and the emphasis on co-cultivation of various microorganisms to produce novel bioactive molecules are some of the challenges (**Figure 1**). The industrial availability of these compounds is currently limited by low fermentation yields and challenging accessibility via synthesis, necessitating the development of genetically modified strains and improved cultivation techniques (Kaspar et al., 2019).

Cross-feeding is the metabolic exchange that occurs when one bacterial species creates metabolites that another can use within the gut. This complicated interplay between microbial communities can substantially impact comprehending the microbiome’s overall dynamics. Cross-feeding contributes a layer of complexity to microbiome research by influencing multiple species’ growth, survival, and metabolic activity in ways that are not immediately apparent when analyzing individual bacterial relationships (Culp and Goodman, 2023).

## 4 Gut bacterial metabolite interactions

**Figure 2:**
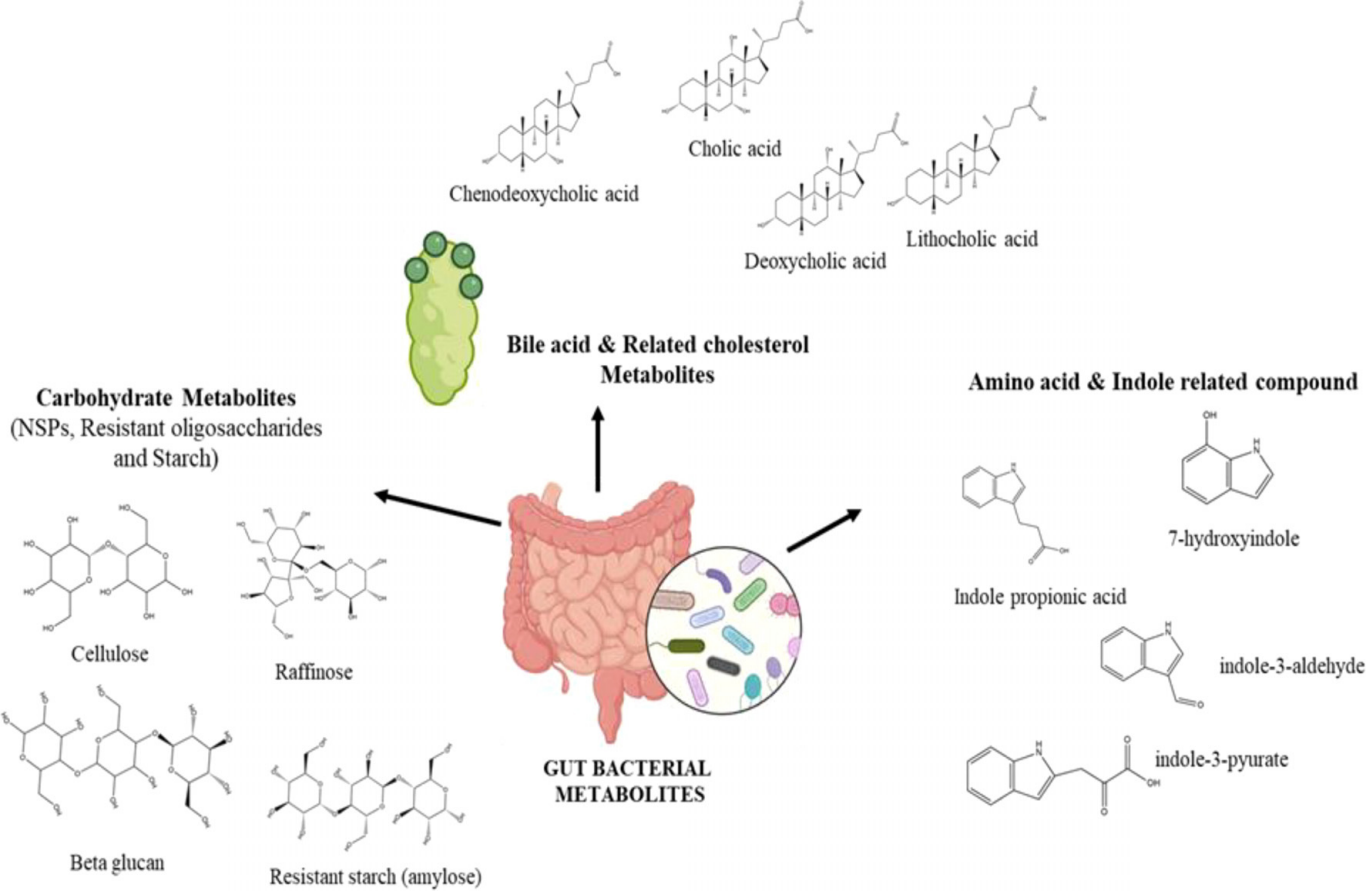
Different types of gut metabolites produced by bacteria.

Since its discovery, numerous studies have highlighted the role of microbiota in health and diseases. Microbiota can be divided into four categories: gut, oral, respiratory, and skin microbiota, depending on the specific areas (Hou and Majumder, 2021). The microbiota significantly shapes the host immune system, affecting how it is trained, activated, and functions. As a result, the immune system has developed to coexist harmoniously with these various microorganisms. This partnership makes it possible to mount defences against infections and to regulate tolerance to harmless antigens (Belkaid and Hand, 2014). Major groups of microbial metabolites include carbohydrate metabolites, amino acid and related metabolites, and lipid and bile acid metabolites (**Figure 2**). Bacteria in our digestive tract convert carbohydrates into SCFAs, such as butyrate, propionate, and acetate. The non-digestive carbohydrates such as non-starch polysaccharides (NSPs), resistant oligosaccharides, and starch are used as substrates by colonic microbes, transforming them into various metabolites including SCFAs (Cronin et al., 2021). The insoluble NSPs, such as cellulose and hemicellulose, are effective laxatives. In contrast, soluble NSPs, such as mixed-link beta-glucans, reduce plasma cholesterol levels and help normalize blood glucose and insulin levels. Polysaccharides such as these can be incorporated into dietary plans to treat cardiovascular disease and type 2 diabetes (Kumar et al., 2012). In particular, short-chain fatty acids promote effective large bowel function and promoting health.

Soluble fibers and resistant starches are fermented to produce these (butyrate, propionate, and acetate) advantageous fatty acids. The capacity of different gut bacteria to produce SCFAs varies. After being created, SCFAs are taken up by gut cells and other cells, where they interact with particular surface receptors. These receptors, like GPR43 and GPR41, are responsible for the various effects SCFAs have on our bodies (Henrissat, 1991). They are important energy sources for colonocytes, affect the function of the epithelial barrier, and control both innate and adaptive immune cells (Parada Venegas et al., 2019). As tumor suppressors, SCFAs promote intestinal epithelial cell growth, impede histone deacetylase (HDACs) activity, and cause the differentiation and apoptosis of cancer cells. SCFAs can encourage the hyperproliferation of some colon epithelial cells, which may contribute to the development of tumors, so caution is advised. Mucin production, barrier integrity, and epithelial cytokine production are all positively impacted by SCFAs. They control the functional activities of dendritic cells (DCs), macrophages, and neutrophils. Additionally, SCFAs enhance the populations of regulatory and effector T-cells by promoting B-cell production of antibodies and modulating T-cell differentiation. However, depending on concentration, organ, and host state, SCFAs can also elicit inflammatory responses despite their positive effects. SCFAs are essential for immune regulation and inflammation (Sakata and Von Engelhardt, 1983). Gut bacteria break down leftover proteins from our diet, producing various metabolites. These include branched-chain SCFAs, ammonia, and biogenic amines. Particularly, the metabolism of tryptophan produces indole, which can activate cellular pathways such as AhR and PXR (Macfarlane et al., 1988).

Aryl hydrocarbon receptor (AhR) activation is a major function of major indole derivatives derived from tryptophan or plant-derived glucobrassicin in the immune regulatory functions of amino acid and indole-related metabolites. AhR reacts to a variety of natural and artificial substances, affecting immune responses that are modulated by environmental pollutants as well as those that are controlled by dietary factors. AhR translocate to the nucleus upon ligand binding and forms a complex with AhR nuclear translocator. The xenobiotic responsive element in genes that code for immune-regulatory molecules or toxicant-modifying enzymes is bound by this complex. Many genes, such as those for CYP450 1A1, IDO, IL-10, Aiolos, FoxP3, IL-21, and CD39, are regulated by AhR. The way that AhR ligands affect T-cell responses is complex, depends on different cell types and factors, and isn’t always the same as how AhR functions in immune responses as a whole (Apetoh et al., 2010). Polyamines have anti-inflammatory properties and control transcription, translation of proteins, responses of stress proteins, and cellular metabolism. They help to repair tissue damage while suppressing inflammatory T-cells, cytokine production, and nitric oxide (NO). It is still unknown, though, how gut microbiota-derived polyamines cross the gut barrier to control immune cells at the molecular level (Keough et al., 2011).

Through enzymatic processes, gut bacteria modify bile acids and dietary lipids. New molecules are produced by these reactions, such as secondary bile acids and hydroxy fatty acids. It’s interesting to note that these bacterial metabolites are more than just waste materials; they can also interact with particular cell receptors like FXR, VDR, PXR, and TGR5. These receptor interactions play a crucial role in regulating various cellular processes within our body (Polan et al., 1964; Kishino et al., 2013). Although bile acids have anti-inflammatory properties and are important in cholestatic and metabolically driven inflammatory diseases, long-term exposure to high levels of bile acids can cause cancer and inflammation. Secondary bile acids control the immune system through receptors like TGR5, FXR, and PXR, such as lithocholic acid and deoxycholic acid. Animals with more severe colitis exhibit deficiencies in these receptors, indicating a potential function for these receptors in immune tolerance promotion. TGR5 ligands inhibit nuclear factor kappa B (NF-*κ*B) by inducing cAMP synthesis, suppressing TNF-α production, and phosphorylating c-Fos. The NF-*κ*B is a transcription factor that plays a role in many biological processes, including immune response and inflammation. Additionally, DCs produced by TGR5 ligands produce less IL-12 (Gerard, 2013; Kishino et al., 2013).

## 5 Future directions and conclusions

Metagenomics is a new player in this field, which combines mass spectrometry-based metabolomics research with genome sequencing and automated gene cluster annotation. The discovery of previously unidentified physiologically active compounds resulted from research focusing on the relationship between genes and metabolites or metabolites and genes. Identifying new secondary metabolites and concurrent redesign of established biosynthetic pathways to improve the synthesis of novel compounds will be aided by integrating genes to secondary metabolites and secondary metabolites to genes employing a forward or retro-biosynthetic approach. The immune regulatory roles of specific metabolites in health and disease need to be further investigated. More importantly, it is still unclear how the combination of these microbial metabolites affects the host immune system. In addition, research on the relationship between altered gut microbial metabolite composition and immune responses and particular diseases is necessary to find pathological condition biomarkers. In conclusion, despite the potential that bacterial metabolites have as immunomodulators and for the treatment of immune-related illnesses, several obstacles and restrictions prevent their broad application. These include the intricacy and diversity of bacterial metabolites, our incomplete knowledge of their mechanisms of action, problems with their stability, production, and purification, as well as safety, immune tolerance, and off-target effects concerns. To overcome these challenges and fully realize the therapeutic potential of bacterial metabolites in precision medicine approaches for immune-related disorders, collaborative interdisciplinary research efforts are essential.
